# Small field output correction factors of the microSilicon detector and a deeper understanding of their origin by quantifying perturbation factors

**DOI:** 10.1002/mp.14149

**Published:** 2020-04-13

**Authors:** Carolin Weber, Rafael Kranzer, Jan Weidner, Kevin Kröninger, Björn Poppe, Hui Khee Looe, Daniela Poppinga

**Affiliations:** ^1^ PTW Freiburg Freiburg 79115 Germany; ^2^ TU Dortmund University Dortmund 44227 Germany; ^3^ University Clinic for Medical Radiation Physics Medical Campus Pius Hospital Carl von Ossietzky University Oldenburg 26121 Germany

**Keywords:** density, detector response, Monte Carlo, perturbation factor, small field

## Abstract

**Purpose:**

The aim of this study is the experimental and Monte Carlo‐based determination of small field correction factors for the unshielded silicon detector microSilicon for a standard linear accelerator as well as the Cyberknife System. In addition, a detailed Monte Carlo analysis has been performed by modifying the detector models stepwise to study the influences of the detector’s components.

**Methods:**

Small field output correction factors have been determined for the new unshielded silicon diode detector, microSilicon (type 60023, PTW Freiburg, Germany) as well as for the predecessors Diode E (type 60017, PTW Freiburg, Germany) and Diode SRS (type 60018, PTW Freiburg, Germany) for a Varian TrueBeam linear accelerator at 6 MV and a Cyberknife system. For the experimental determination, an Exradin W1 scintillation detector (Standard Imaging, Middleton, USA) has been used as reference. The Monte Carlo simulations have been performed with EGSnrc and phase space files from IAEA as well as detector models according to manufacturer blueprints. To investigate the influence of the detector’s components, the detector models have been modified stepwise.

**Results:**

The correction factors for the smallest field size investigated at the TrueBeam linear accelerator (equivalent dosimetric square field side length S_clin_ = 6.3 mm) are 0.983 and 0.939 for the microSilicon and Diode E, respectively. At the Cyberknife system, the correction factors of the microSilicon are 0.967 at the smallest 5‐mm collimator compared to 0.928 for the Diode SRS. Monte Carlo simulations show comparable results from the measurements and literature.

**Conclusion:**

The microSilicon (type 60023) detector requires less correction than its predecessors, Diode E (type 60017) and Diode SRS (type 60018). The detector housing has been demonstrated to cause the largest perturbation, mainly due to the enhanced density of the epoxy encapsulation surrounding the silicon chip. This density has been rendered more water equivalent in case of the microSilicon detector to minimize the associated perturbation. The sensitive volume itself has been shown not to cause observable field size‐dependent perturbation except for the volume‐averaging effect, where the slightly larger diameter of the sensitive volume of the microSilicon (1.5 mm) is still small at the smallest field size investigated with corrections <2%. The new microSilicon fulfils the 5% correction limit recommended by the TRS 483 for output factor measurements at all conditions investigated in this work.

## 1 Introduction

Modern radiotherapy techniques are based on highly modulated treatment deliveries by superposing the dose distributions from very small field sizes down to a few millimeters to achieve high‐dose conformation. The use of small field sizes is also one of the prerequisites in stereotactic radiotherapy, where small lesions are irradiated with a high dose per fraction and steep dose falloff to achieve better sparing of organs at risk. Nevertheless, the dosimetry of these small field sizes is associated with different challenges, such as the lack of lateral secondary electronic equilibrium and its implications on the response of detectors having finite dimensions and constructed from non‐water‐equivalent materials.^1^, ^2^, ^3^, ^4^, ^5^, ^6^, ^7^, ^8^ These aspects have been addressed in different dedicated protocols, including the codes of practice published recently by the IAEA as TRS 483.^9^ Besides providing clinical guidelines for small field dosimetry, the TRS 483 also serves the purpose of standardizing the reporting of correction factors, such as the small field output correction factors $k_{Q_{\mathrm{clin}},Q_{\mathrm{msr}}}^{f_{\mathrm{clin}},f_{\mathrm{msr}}}$ proposed by Alfonso et al.^10^ For the common detectors used in small field dosimetry, the TRS 483 has also recommended the corresponding factors $k_{Q_{\mathrm{clin}},Q_{\mathrm{msr}}}^{f_{\mathrm{clin}},f_{\mathrm{msr}}}$ based on the literature available during the compilation. These factors, however, must be updated when new data or new detectors become available, for which no or limited data is published.

Serving the purpose of IAEA TRS 483, the first aim of this work is to determine the small field correction factors $k_{Q_{\mathrm{clin}},f_{\mathrm{msr}}}^{f_{\mathrm{clin}},f_{\mathrm{msr}}}$ for a new unshielded silicon diode detector, microSilicon (type 60023, PTW Freiburg, Germany), which is the successor of two unshielded diode detectors, Diode E (type 60017, PTW Freiburg, Germany) and Diode SRS (type 60018, PTW Freiburg, Germany). The correction factors were derived conforming to the TRS 483 formalism for a conventional linear accelerator and a Cyberknife System. For both systems, the correction factors were determined experimentally and by Monte Carlo (MC) simulations.

To better comprehend the differences between the new microSilicon detector and its predecessors, the second aim of this work is to perform a detailed analysis on the roles of detector construction on the small field perturbation effects, such as the volume‐averaging and density effects. Using Monte Carlo methods, these effects were separated and discussed for the different detector types.

## 2 Materials and Methods

### 2.A Detectors

The new microSilicon detector (type 60023, PTW Freiburg, Germany) was investigated along with its two predecessors, Diode E (type 60017, PTW Freiburg, Germany) and Diode SRS (type 60018, PTW Freiburg, Germany). The active volume of the microSilicon has a slightly larger diameter of 1.5 mm compared to 1.2 mm of its predecessors.^11^ Furthermore, the density of the surrounding casting compound material has been reduced significantly to a density of 1.15 g/cm^3^ to achieve a more water‐equivalent characteristic.

### 2.B Formalism of small field dosimetry

The determination of small field output correction factors in this wok was performed according to the formalism proposed by Alfonso et al.^10^ and later adopted in the TRS 483. The deviation of a detector’s dose response in a clinical field $f_{\mathrm{clin}}$ from its dose response in a machine‐specific reference field $f_{\mathrm{msr}}$ is corrected by the factor $k_{Q_{\mathrm{clin}},Q_{\mathrm{msr}}}^{f_{\mathrm{clin}},f_{\mathrm{msr}}}$ defined as

(1) $$k_{Q_{\mathrm{clin}},Q_{\mathrm{msr}}}^{f_{\mathrm{clin}},f_{\mathrm{msr}}}=\frac{D_{w,Q_{\mathrm{clin}}}^{f_{\mathrm{clin}}}/M_{Q_{\mathrm{clin}}}^{f_{\mathrm{clin}}}}{D_{w,Q_{\mathrm{msr}}}^{f_{\mathrm{msr}}}/M_{Q_{\mathrm{msr}}}^{f_{\mathrm{msr}}}}$$

$D_{w,Q_{\mathrm{clin}}}^{f_{\mathrm{clin}}}$ describes the absorbed dose to water at a clinical field $f_{\mathrm{clin}}$ of quality $Q_{\mathrm{clin}}$, whereas $D_{w,Q_{\mathrm{msr}}}^{f_{\mathrm{msr}}}$ is the absorbed dose to water at the machine‐specific reference field $f_{\mathrm{msr}}$ of quality $Q_{\mathrm{msr}}$. $M_{Q_{\mathrm{clin}}}^{f_{\mathrm{clin}}}$ and $M_{Q_{\mathrm{msr}}}^{f_{\mathrm{msr}}}$ are the detector readings at $f_{\mathrm{clin}}$ and $f_{\mathrm{msr}}$, respectively, corrected for the influence quantities such as temperature, pressure, recombination, and polarity effect.

On the one hand, the detector output ratio is defined as

(2) $$OR_{\det}=\frac{M_{Q_{\mathrm{clin}}}^{f_{\mathrm{clin}}}}{M_{Q_{\mathrm{msr}}}^{f_{\mathrm{msr}}}}$$

which considers the difference of the detector readings between $f_{\mathrm{clin}}$ and $f_{\mathrm{msr}}$. On the other hand, the field output factor, which is the ratio of the absorbed dose to water at *f*_clin_, and absorbed dose to water at *f*_msr_ is given by

(3) $$\Omega_{Q_{clin,}Q_{\mathrm{msr}}}^{f_{cjlin,}f_{\mathrm{msr}}}=\frac{D_{w,Q_{\mathrm{clin}}}^{f_{\mathrm{clin}}}}{D_{w,Q_{\mathrm{msr}}}^{f_{\mathrm{msr}}}}$$

Combining Eqs. (1), (2), and (3), the correction factor $k_{Q_{\mathrm{clin}},Q_{\mathrm{msr}}}^{f_{\mathrm{clin}},f_{\mathrm{msr}}}$ can be written as

(4) $$k_{Q_{\mathrm{msr}}f_{\mathrm{msr}}}^{Q_{\mathrm{clin}}f_{\mathrm{clin}}}=\frac{\Omega_{Q_{clin,}Q_{\mathrm{msr}}}^{f_{cjlin,}f_{\mathrm{msr}}}}{OR_{\det}}$$

### 2.C Measurements

The measurements were performed at a TrueBeam linear accelerator (Varian Medical Systems, Palo Alto, USA) and at a Cyberknife M6 system (Accuray, Sunnyvale, USA) using an MP3 phantom (PTW Freiburg, Germany). For the Truebeam measurements, nominal field sizes, defined by jaws, with field side lengths of [0.6, 0.8, 1.0, 1.2, 1.5, 2, 4, 10] cm were used and the microSilicon detector was compared to the Diode E detector. In the case of the Cyberknife system, circular fixed collimators with diameter of [0.5, 0.75, 1.0, 1.25, 1.5, 2, 6] cm were used and the microSilicon detector was compared to the Diode SRS detector. The detectors were positioned axially with their effective points of measurement at the measurement depth using the Trufix positioning system (PTW Freiburg, Germany). The output measurements at the Truebeam linear accelerator were performed in 10‐cm water depth with source‐to‐surface distance (SSD) of 90 cm. The output measurements at the Cyberknife system were performed at 1.5‐cm water depth with an SSD of 78.5 cm. A Tandem electrometer and the software package CenterCheck were used to position the detectors at the center of each radiation field. The equivalent dosimetric square field sizes corresponding to the nominal field sizes at the Truebeam linear accelerator were derived from profiles measured with a microDiamond detector (type 60019, PTW Freiburg, Germany).

**Fig. 1:**
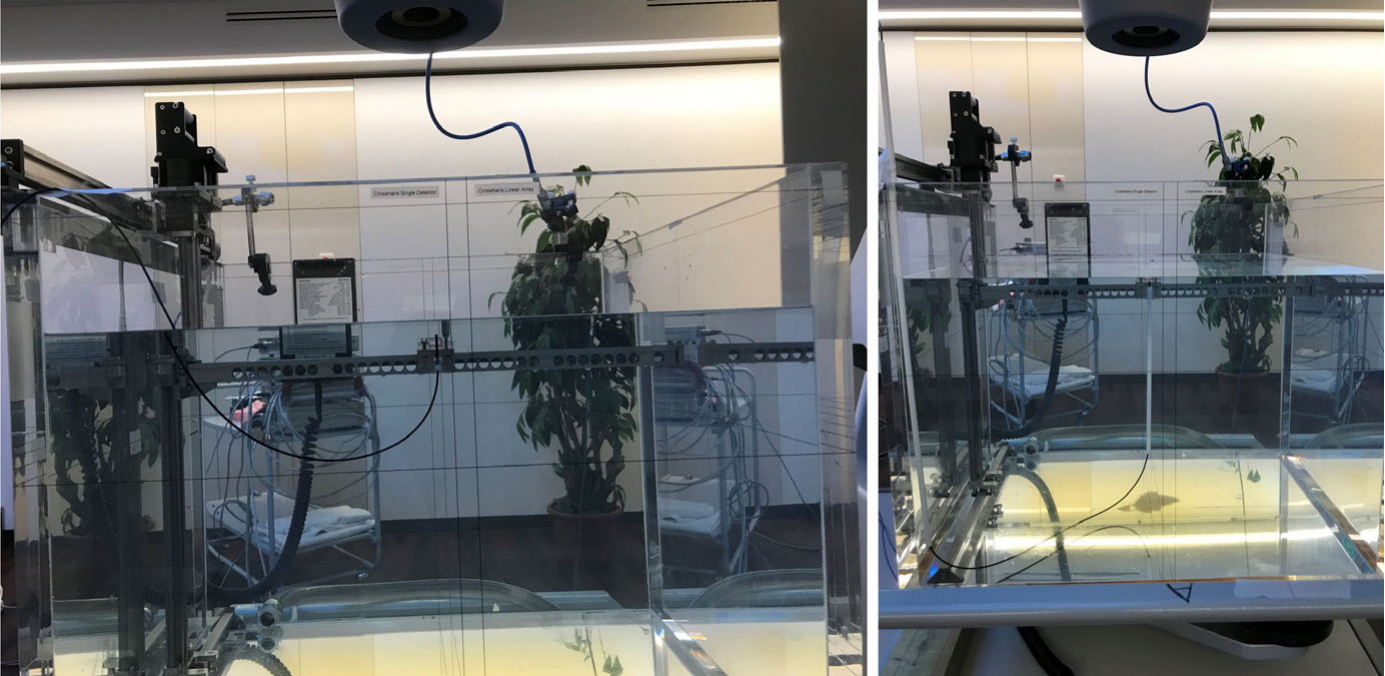
Measurement setup to obtain the Cherenkov light ratio values. Left: minimum fiber configuration; right: maximum fiber configuration. [Color figure can be viewed at wileyonlinelibrary.com]

A plastic scintillation detector W1 (Standard Imaging, Middleton, USA) was used as the reference detector to obtain the field output factor. The correction of Cherenkov signal in the stem was performed according to the method described in Ref. [12, 13, 14]: The detector was positioned axially and irradiated with a 10 cm × 10 cm field at the Truebeam linear accelerator, and with a 6‐cm diameter field at the Cyberknife system. The minimum fiber configuration was realized by directing the fiber perpendicular to the beam's axis out of the field and phantom, while the maximum fiber configuration was achieved by guiding the fiber along the beam’s axis down to the bottom of the phantom with a fiber holder connected to the TRUFIX system. Both configurations are shown in Fig. 1. The maximum fiber configuration was used for the measurement of the output ratio values, since in this configuration, the variation of the irradiated fibers for different field sizes can be minimized. The W1 signal was read out using two UNIDOS webline electrometer systems (PTW Freiburg, Germany) with 1 fA resolution positioned outside the treatment room. The integration time for each measurement was 120 s. The Cherenkov Light Ratio (CLR) values were determined to be 0.696 at the Cyberknife system and 0.657 at the Truebeam linear accelerator according to the method described by Morin et al.^14^

### 2.D Monte Carlo simulations

The Monte Carlo toolkit *EGSnrc* ^15^ with the user code *egs_chamber* ^16^ was used in this study. Global simulation cutoff energies of 521 and 10 keV were chosen for electrons and photons, respectively. The *photon cross section enhancement* (XCSE) was used with a cross section enhancement factor of 128 within a box of 1 × 1 × 1 cm^3^ surrounding the whole detector geometry. Furthermore, the *range rejection* with a rejection factor of 256 and an energy limit $E_{\mathrm{save}}$ of 521 keV was chosen. The size of the phantom modeled was 30 × 30 × 30 cm^3^. The relevant MC parameters are summarized in Table I recommended by AAPM Task Group Report 268.^17^

**Table I:** Monte Carlo parameters.

Parameter	Description	References
Code, version/release date	EGSnrc/usercodeegs_chamber Version 4, released on 18 Apr 2018	Kawrakow et al.^15^ ; Wulff et al.^16^
Electron‐step algorithm	EGSnrc/PRESTA‐II	
Validation		Kawrakow et al.^15^
Source description	IAEA phase space files for a Varian ClinaciX 6 MV and CyberKnife system	Download: www‐nds.iaea.org/phsp Varian ClinaciX^18^ Cyberknife^19^
Bremsstrahlung cross sections	BH (default)	
Photon cross sections	XCOM	
Brems angular sampling	KM (default)	
Spin Effects	On (default)	
Electron impact ionization	Off (default)	
Rayleigh scattering	On (default)	
Bound compton scattering	Norej (default)	
Radiative compton corrections	Off (default)	
Atomic relaxations	eadl	
Pair angular sampling	Simple	
Triplet production	Off (default)	
Photoelectron angular sampling	On (default)	
Photonuclear attenuation	Off	
Boundary crossing algorithm	Exact	
Skin depth for BCA	3 (default)	
Threshold kinetic energy inelastic collisions	AE=521keV	
Threshold energy radiative collisions	AP=1keV own defined media AP=10keV predefined media	
Charged particle cutoff	ECUT=521keV	
Photon cutoff energy	PCUT=10keV	
Photon cross section enhancement	XCSEfactor=128 within a box of 1cm side length	Wulff et al.^16^
Electron range rejection	$E_{save}=521keV$ RangeRejectionfactor=256	
Scored quantities	Dose in the sensitive volume	
# histories/statistical uncertainty	For each field size 1000 runs with different random seed each using the number of particles in the related phase space file	
Statistical methods	History‐by‐history	Sempau et al.^23^
Postprocessing	Dose from output files is taken directly for calculation of correction and perturbation factors [Eqs. (1), (5), (6), and (7)] with Python	

The simulations of small field output correction factors $k_{Q_{\mathrm{clin}},Q_{\mathrm{msr}}}^{f_{\mathrm{clin}},f_{\mathrm{msr}}}$ were performed using the phase space data files downloaded from the IAEA database (www‐nds.iaea.org/phsp) of a Varian Clinac iX (www‐nds.iaea.org/phsp/photon/Varian_Clinac_iX_6MV/) and a CyberKnife system (www‐nds.iaea.org/phsp/photon/CyberKnife_IRIS/). These phase space data have already been used by several other publications.^18^, ^19^, ^20^, ^21^, ^22^ The data of the 6‐MV Varian Clinac iX model provide nominal field sizes of 0.5 × 0.5 cm^2^, 1 × 1 cm^2^, 2 × 2 cm^2^, and 4 × 4 cm^2^, where the 4 × 4 cm^2^ was taken as the msr field, $f_{\mathrm{msr}}$. The simulations were performed with SSD 100 cm according to the scoring plane in the phase space files. For the Cyberknife system, phase space files of the iris collimators with nominal diameters of 0.5, 0.75, 1, 1.5, and 6 cm were used, whereas the largest diameter of 6 cm serves as the msr field, $f_{\mathrm{msr}}$, as recommend by TRS 483. For each phase space file, the effective dosimetric field size S_clin_ at the detector position was calculated and summarized in Table II. The overall uncertainty of the Monte Carlo simulations in this study was calculated considering both the statistical uncertainty and the latent variance of the phase space files evaluated using the method described in Sempau et al.^23^

**Table II:** Numerical values of Fig. 3. The uncertainty (k = 1) of the correction factors given in the table is +0.010 for the experimental values and +0.006 for the Monte Carlo‐based values.

Varian TrueBeam							
Diode E 60017				microSilicon 60023			
Experiment		Monte Carlo		Experiment		Monte Carlo	
Equivalent field size length Sclin/cm	$k_{Q_{\mathrm{clin}}{,Q}_{4x4}}^{f_{\mathrm{clin}}{,f}_{4x4}}$	Equivalent field size length Sclin/cm	$k_{Q_{\mathrm{clin}}{,Q}_{4x4}}^{f_{\mathrm{clin}}{,f}_{4x4}}$	Equivalent field size length Sclin/cm	$k_{Q_{\mathrm{clin}}{,Q}_{4x4}}^{f_{\mathrm{clin}}{,f}_{4x4}}$	Equivalent field size length Sclin/cm	$k_{Q_{\mathrm{clin}}{,Q}_{4x4}}^{f_{\mathrm{clin}}{,f}_{4x4}}$
0.63	0.939	0.58	0.930	0.63	0.983	0.58	0.979
0.82	0.956			0.82	0.986		
1.01	0.973	1.08	0.979	1.01	0.991	1.08	0.993
1.50	0.992			1.50	1.000		
2.00	1.004	2.17	0.999	2.00	1.004	2.17	1.004
3.00	1.002			3.00	1.002		
4.00	1.000	4.39	1.000	4.00	1.000	4.39	1.000
10.00	0.986			10.00	0.989		

Cyberknife							
Diode SRS 60018				microSilicon 60023			
Experiment		Monte Carlo		Experiment		Monte Carlo	
Field size length/cm	$k_{Q_{\mathrm{clin}}{.Q}_{6cm}}^{f_{\mathrm{clin}}{,f}_{6cm}}$	Equivalent field size length S_clin_/cm	$k_{Q_{\mathrm{clin}}{,Q}_{6cm}}^{f_{\mathrm{clin}}{,f}_{6cm}}$	Field size length/cm	$k_{Q_{\mathrm{clin}}{,Q}_{6cm}}^{f_{\mathrm{clin}}{,f}_{6cm}}$	Equivalent field size length S_clin_/cm	$k_{Q_{\mathrm{clin}}{,Q}_{6cm}}^{f_{\mathrm{clin}}{,f}_{6cm}}$
0.5	0.928	0.52	0.940	0.5	0.967	0.52	0.970
0.75	0.960	0.72	0.956	0.75	0.980	0.72	0.976
1.0	0.971	0.95	0.970	1.0	0.985	0.95	0.982
1.25	0.985			1.25	0.992		
1.5	0.993	1.43	0.993	1.5	0.996	1.43	0.993
2.0	1.002			2.0	1.003		
6.0	1.000	57.1	1.000	6.0	1.000	57.1	1.000

**Fig. 2:**
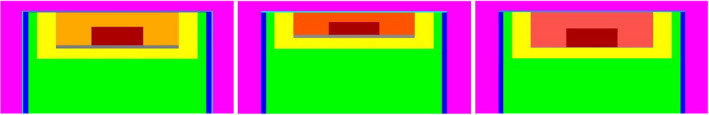
Monte Carlo detector models. Left: Diode E type 60017 center: Diode SRS type 60018 right: microSilicon type 60023. Identical colors indicate identical materials. [Color figure can be viewed at wileyonlinelibrary.com]

Detailed detector models were implemented following the manufacturer’s blueprints as shown in Fig. 2. For each detector’s geometry, a Fano test was performed using the egs_fano_source as described in Czarnecki et al.^24^ The results from the Fano tests are summarized in the Appendix.

The detectors were positioned as during the measurements, that is, in 10‐cm water depth and an SSD of 90 cm for the Varian Clinac iX simulations; and in 1.5‐cm water depth and an SSD of 78.5 cm for the Cyberknife system, within a water phantom of 50 × 50 × 35 cm^3^.

The output ratios, *OR_det_*, were determined according to Eq. (2) by simulating the dose deposited inside the sensitive volumes of the detectors. The field output factors in Eq. (3), $\Omega_{Q_{clin,}Q_{\mathrm{msr}}}^{f_{cjlin,}f_{\mathrm{msr}}}$, were calculated by scoring the dose in a small cylindrical water voxel with a radius of 0.5 mm and 0.05 mm height at the same point of measurement (comparable to Francescon et al.^25^). The correction factors, $k_{Q_{\mathrm{msr}}f_{\mathrm{msr}}}^{Q_{\mathrm{clin}}f_{\mathrm{clin}}}$, were then calculated according to Eq. (4).

**Fig. 3:**
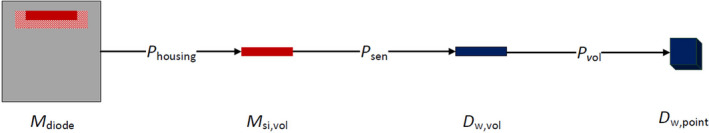
Simulation geometries used for the decomposition of the small field output correction factors to quantify the perturbations caused by different detector’s components. The model of *M*_Diode_ is the same as the one used in the simulations of the correction factors $k_{Q_{\mathrm{clin}},Q_{\mathrm{msr}}}^{f_{\mathrm{clin}},f_{\mathrm{msr}}}$ ^.^ [Color figure can be viewed at wileyonlinelibrary.com]

To understand the influences of different detector components on the total correction factors, detailed simulations were performed by modifying the models of the microSilicon and Diode E detectors stepwise as shown in Fig. 3. In the first modification, the complete detector’s housing was substituted by water, except the sensitive silicon volume, to obtain *M*_si,vol_. In the second modification, the sensitive silicon volume was replaced with water to obtain *D*_w,vol_. In the last step, the dose within a small water cylinder, *D*_w,point_, corresponds to the previous simulations of field output factors. The simulations were performed using the Varian Clinac iX phase space files of field sizes of 0.5 × 0.5 cm^2^, 1 × 1 cm^2^, 2 × 2 cm^2^, and 4 × 4 cm^2^ as well as the Cyberknife phase space files of field sizes of [0.5, 0.75, 1, 1.5, 6] cm. The influence of each detector component was characterized using the corresponding field size‐dependent perturbation factors:

(5) $$P_{\mathrm{housing}}^{f_{\mathrm{clin}},f_{\mathrm{msr}}}=\frac{M_{\text{si},\text{vol}}^{f_{\text{clin}}}/M_{\mathrm{diode}}^{f_{\text{clin}}}}{M_{\text{si},\text{vol}}^{f_{\text{msr}}}/M_{\mathrm{diode}}^{f_{\text{msr}}}}$$

(6) $$P_{\mathrm{sens}}^{f_{\mathrm{clin}},f_{\mathrm{msr}}}=\frac{D_{w,\text{vol}}^{f_{\text{clin}}}/M_{si,vol}^{f_{\text{clin}}}}{D_{w,\text{vol}}^{f_{\text{msr}}}/M_{si,vol}^{f_{\text{msr}}}}$$

(7) $$P_{\mathrm{vol}}^{f_{\mathrm{clin}},f_{\mathrm{msr}}}=\frac{D_{w,\text{point}}^{f_{\text{clin}}}/D_{w,vol}^{f_{\text{clin}}}}{D_{w,\text{point}}^{f_{\text{msr}}}/D_{w,vol}^{f_{\text{msr}}}}$$

*P*_housing_ represents the perturbation caused by the housing around the active volume, which means outer capsulation, housing of the chip, casting compound material, and the volume of the silicon chip, which is not the active detector volume. *P*_sen_ represents the perturbation caused by the difference of the material of the active volume compared to water and *P*_vol_ accounts for the finite dimension of the active volume compared to an infinitesimal water voxel according to *D*_w,point_.

## 3 Results

**Fig. 4:**
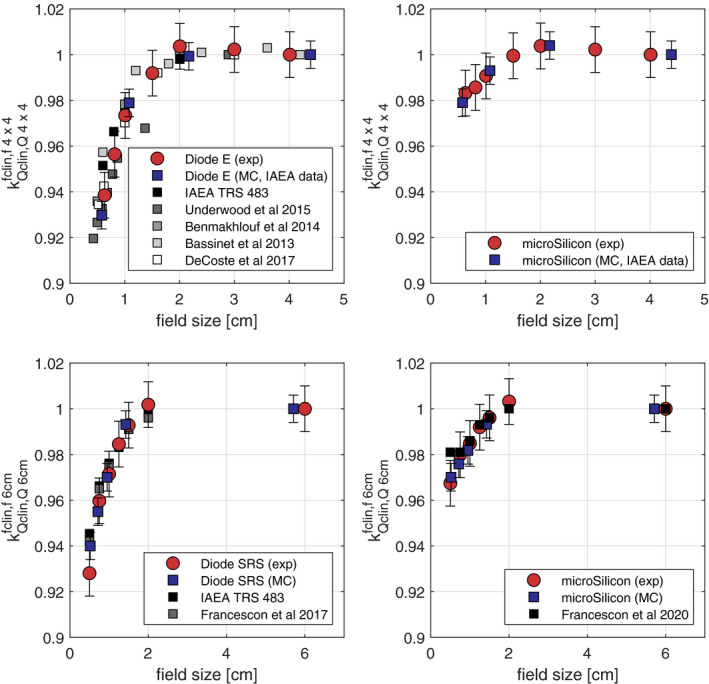
Measured and simulated correction factors, $k_{Q_{\mathrm{clin}},Q_{\mathrm{msr}}}^{f_{\mathrm{clin}},f_{\mathrm{msr}}}$, obtained for Varian linear accelerator using (a) Diode E and (b) microSilicon; measured and simulated correction factors, $k_{Q_{\mathrm{clin}},Q_{\mathrm{msr}}}^{f_{\mathrm{clin}},f_{\mathrm{msr}}}$, obtained for Cyberknife system using (c) Diode SRS and (d) microSilicon. Diode E and Diode SRS results are compared to different publications.^9^, ^20^, ^26^, ^32^, ^33^, ^34^, ^35^ [Color figure can be viewed at wileyonlinelibrary.com]

The measured correction factors, $k_{Q_{\mathrm{msr}},Q_{\mathrm{msr}}}^{f_{\mathrm{clin}},f_{\mathrm{msr}}}$, for the Varian TrueBeam linear accelerator using the Diode E and the microSilicon are shown in Figs. 4(a) and 4(b), respectively. The Monte Carlo simulated $k_{Q_{\mathrm{clin}},Q_{\mathrm{msr}}}^{f_{\mathrm{clin}},f_{\mathrm{msr}}}$ using the IAEA phase spaces files for a Varian Clinac iX linear accelerator are presented for comparison. Both the measured and simulated correction factors of the Diode E are also compared to the recommended values in the TRS 483 and the recent data available in the literature obtained at Varian linear accelerators. Both diode detectors show over‐response at field sizes below 2 cm, that is, the correction factors are less than unity. At the smallest field size, the Diode E requires correction up to 8%. The required correction for the new microSilicon is significantly lower with a maximum correction of around 2% at the smallest field size investigated.

The measured and simulated correction factors $k_{Q_{\mathrm{clin}},Q_{\mathrm{msr}}}^{f_{\mathrm{clin}},f_{\mathrm{msr}}}$ for the Cyberknife system are presented in Figs. 4(c) and 4(d). Similar to the results of Varian linear accelerators, the microSilicon requires less corrections than the Diode SRS. At the smallest field size, the Diode SRS requires correction up to 6%, whereas the microSilicon requires correction of around 3%. Therefore, the required corrections of the microSilicon are less than the 5% limit recommended by the TRS 483 for all the investigated conditions in this work. The numerical values of the correction factors are given in the Table II.

**Table III:** Parameters used for the detectors’ models in this study.

Component	Material			Density $\left(\frac{g}{cm^{3}}\right)$		
	60017	60018	60023	60017	60018	60023
Detector housing (pink)	RW3	RW3	RW3	1.045	1.045	1.045
Detector housing (light blue)	Ag	Ag	Air	10.50	10.50	1.248e‐3
Detector housing (dark blue)	Al	Al	Al	2.82	2.82	2.82
Detector housing (green)	PEEK	PEEK	PEEK	1.31	1.31	1.31
Detector housing (yellow)	FR4	FR4	FR4	2.00	2.00	2.00
Detector housing (gray)	Cu/Carbon	Cu/Carbon	Cu/Carbon	1.27	1.27	1.27
Epoxy encapsulation (orange shades)	Epoxy	Epoxy	Epoxy	1.77	1.4	1.16
Silicon chip (dark red)	Si	Si	Si	2.33	2.33	2.33

**Fig. 5:**
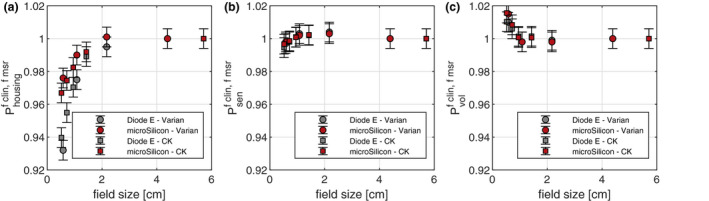
Detector’s components perturbation effect for the Diode E and microSilicon. [Color figure can be viewed at wileyonlinelibrary.com]

The field size‐dependent influence of different detector’s components on the correction factors are demonstrated in Fig. 5. As shown in Fig. 5(a), the housing of the detector represented by *P*_housing_ causes the largest perturbation, mainly due to the epoxy encapsulation of the diode detectors. As shown in Table III and Fig. 2, these epoxy layers surrounding the sensitive volume have density larger than that of normal water, which causes local secondary electron fluence perturbation at small field sizes. As a result, the detector’s dose response will increase with decreasing field sizes. Nevertheless, this perturbation has been reduced for the microSilicon, where the density of the casting compound material has been rendered more water equivalent.

The sensitive volume itself causes no observable perturbation as its thickness merely measures up to 20 μm. The volume‐averaging effect of the microSilicon is slightly higher than that of the Diode E due to the larger diameter of its sensitive volume that measures 1.5 mm compared to 1.2 mm for the Diode E. However, the effect is very small for both detector types and the difference is not statistically significant. There is no difference between Varian Truebeam and Cyberknife simulations.

## 4 Discussion

The applicability of the new microSilicon to measure small field output factors has been compared to its predecessors Diode E and Diode SRS. The W1 scintillation detector has been chosen as the reference detector to obtain the small field output correction factors of the diode detectors. Its suitability to represent the field output factor at small field sizes has been demonstrated repeatedly in the literature^12^, ^26^, ^27^ and TRS 483 defined a small field correction factor of 1. The microSilicon shows lower over‐response compared to Diode E and Diode SRS in small fields at a Varian TrueBeam linear accelerator and a Cyberknife system requiring only corrections of less than 3% for the range of field sizes investigated in this work. Therefore, the recommendation of the recent TRS 483 to only use detectors that require less than 5% corrections in small fields is fulfilled by the microSilicon for the investigated conditions.

Detailed Monte Carlo analysis of the influence of detector’s components on the correction factors $k_{Q_{\mathrm{clin}},f_{\mathrm{msr}}}^{f_{\mathrm{clin}},f_{\mathrm{msr}}}$ has demonstrated that the detector’s housing is the main contributor of the perturbation in small fields. A similar investigation was published by Huet et al.^28^ for the Diode E detector at a Cyberknife System, where they computed three perturbation factors *P*_ext_, *P*_mat_, and *P_v_*_ol_ characterizing the different perturbation components, similar to the approach used in this work. In contrast to this study, their *P*_mat_ accounts for the whole silicon chip, whereas in this study, the *P*_sen_ only represents the perturbation of the active volume and the perturbation of the remaining non‐sensitive part of the silicon chip is included in *P*_housing_. However, our results are comparable to Huet et al.^28^ by comparing the product *P*_housing_ × *P*_sen_ of this study with their product *P*_ext_ × *P*_mat_. At 5‐mm field size, their calculated values of *P*_ext_ and *P*_mat_ of the Diode E are 0.952 and 0.970, respectively, and the product *P*_ext_ × *P*_mat_ = 0.923 is comparable to the product *P*_housing_ × *P*_sen_ = (0.934 ± 0.006) presented in this work.

Furthermore, Looe et al.^4^ have studied the influence of the mass density of surrounding layers upstream and downstream a sensitive layer and found that the electron fluence within the sensitive layer is enhanced with increased mass density of the upstream layer in small fields, where lateral secondary equilibrium is not established. A smaller influence of the downstream layer, in the case of the diode detectors the silicon chip beneath the sensitive volume, has been asserted in the study. Therefore, the underlying mechanism of the observed perturbation due to the detector’s housing can be further traced back to the enhanced density of the epoxy encapsulation surrounding the silicon chip. This modification can be comprehended in Fig. 5(a), where the perturbation factor *P*_housing_ is closer to unity for the microSilicon.

The material of the sensitive volume, in this case silicon (Z = 14) with density 2.32 g/cm^3^, causes no field size‐dependent perturbation. This result agrees to the observation made by Fenwick et al.,^29^, ^30^ who concluded that the influence of the material’s atomic composition on the detector’s dose response is not dependent on the field size. The mass density of the sensitive volume will alter its dose response due to the overshoot or undershoot of the “insiders,” that is, the secondary electrons that are released within the sensitive volume.^1^, ^5^ However, although the mass density of the silicon sensitive volume is higher than normal water, the influence is not observable that can be attributed to its thickness of only 10–20 μm, so that the insiders play only minor contributions to the detector’s response. At the smallest field size investigated, the larger diameter of the microSilicon does not produce significant stronger volume‐averaging effect, where both the Diode E and microSilicon require less than 2% correction. Recently, the influence of the irradiation of cable and other metal contacts on the detector’s signal has been studied^31^ for the PTW microDiamond detector (Type 60019, PTW Freiburg, Germany). It was found that the discrepancy between experimental and Monte Carlo simulated dose response of the microDiamond detector diminishes after accounting for the charge imbalance effect in these components. The silicon detectors investigated in this study have dose response higher than the microDiamond detector by a factor of 10–20. Besides, less metal parts are built in these silicon detectors. Therefore, the role of the cable and metal contacts is considered negligible and hence not considered in this study.

The IAEA phase space files for a Varian Clinac iX and M6 Cyberknife system have been used to simulate the small field output correction factors, $k_{Q_{\mathrm{clin}},Q_{\mathrm{msr}}}^{f_{\mathrm{clin}},f_{\mathrm{msr}}}$, for the three investigated detectors. The Monte Carlo calculated factors for the Diode E using the Clinac iX phase space files are comparable to the correction factors measured at a TrueBeam accelerator and the published values in the literature. In the case of the microSilicon, good agreement was also found between the simulated and measured correction factors. The same applies to the correction factors for Cyberknife system, where the simulated correction factors for the Diode SRS and microSilicon agree with the measured correction factors. It appears that the IAEA phase space files can be utilized in Monte Carlo studies to characterize the detector’s response in small fields and at different machine types. Nevertheless, careful considerations must be made before applying the results due to machine‐to‐machine variabilities and the discrepancies in measurement setup. The available phase space files are valuable to study the underlying physical mechanisms of the observed perturbation effects as has been performed in this study.

## 5 Conclusions

The small field output correction factors, $k_{Q_{\mathrm{clin}},Q_{\mathrm{msr}}}^{f_{\mathrm{clin}},f_{\mathrm{msr}}}$, of the new microSilicon have been determined conforming to the formalism in the TRS 483 for a Varian TrueBeam and a Cyberknife system. Comparisons with its predecessors Diode E and Diode SRS demonstrated that the microSilicon is superior due to a more water‐equivalent design, hence requiring less corrections fulfilling the 5% correction limit recommended by the TRS 483 even at the smallest field size investigated. The data provided in the work will contribute to an updated database for correction factors of the new detector.

## Conflict of Interest

Carolin Weber, Daniela Poppinga, Rafael Kranzer, and Jan Weidner are employees of PTW Freiburg.

## Figures and Tables

**Fig. A1.**
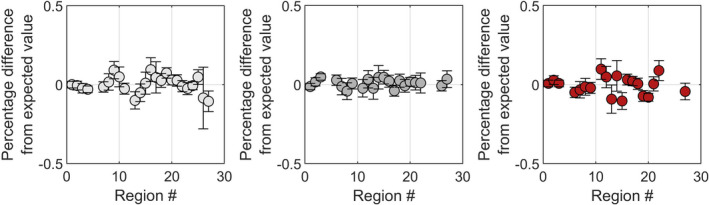
Results of the Fano tests for each detector geometry, uncertainty level of k = 1. Left: Diode E (60017) Center: Diode SRS (60018) Right: microSilicon (60023).

## Appendix 1

The Fano tests have been performed as described by Czarnecki et al.^24^ All materials were replaced by water whereas the density was not changed. A egs_fano_source, which is a uniform isotropic 1.25 MeV monoenergetic electron source, whose intensity scales with the material density, was used. The electron cutoff was chosen as 521 keV and the photon cutoff energy (PCUT) was set to 1.25 MeV to avoid photon transport. The detector models were embedded in a 2 cm × 2 cm × 2 cm water geometry.

The expected dose value is:

(A1) $$Dose_{\mathrm{FANO}}=1.25\;\text{MeV}/m_{\mathrm{geometry}}$$

where m_geometry_ is the mass of the whole Fano source volume.

For each region R, the absorbed dose was determined as

(A2) $$Dose_{R}=Edep_{R}\left(\text{MeV}\right)/m_{R}$$

where m_R_ is the mass of the region under consideration and Edep_ R_ is the energy deposited per history in that region, as reported by EGSnrc.

Under full charge particles equilibrium, one would expect

(A3) $$R_{Region\#}=\left(\frac{Dose_{\mathrm{FANO}}}{Dose_{R}}\right)=1$$

Figure A1 shows the deviations of the computed ratios in Eq. A3 from unity in percent. In all detector regions, the Fano test was passed within ±0.2%.
