# Supplementary figures and images for: Small field output correction factors of the microSilicon detector and a deeper understanding of their origin by quantifying perturbation factors
Source: Med Phys. 2020 Apr 13;47(7):3165–73. doi: 10.1002/mp.14149 (PMC7496769; doi:10.1002/mp.14149)

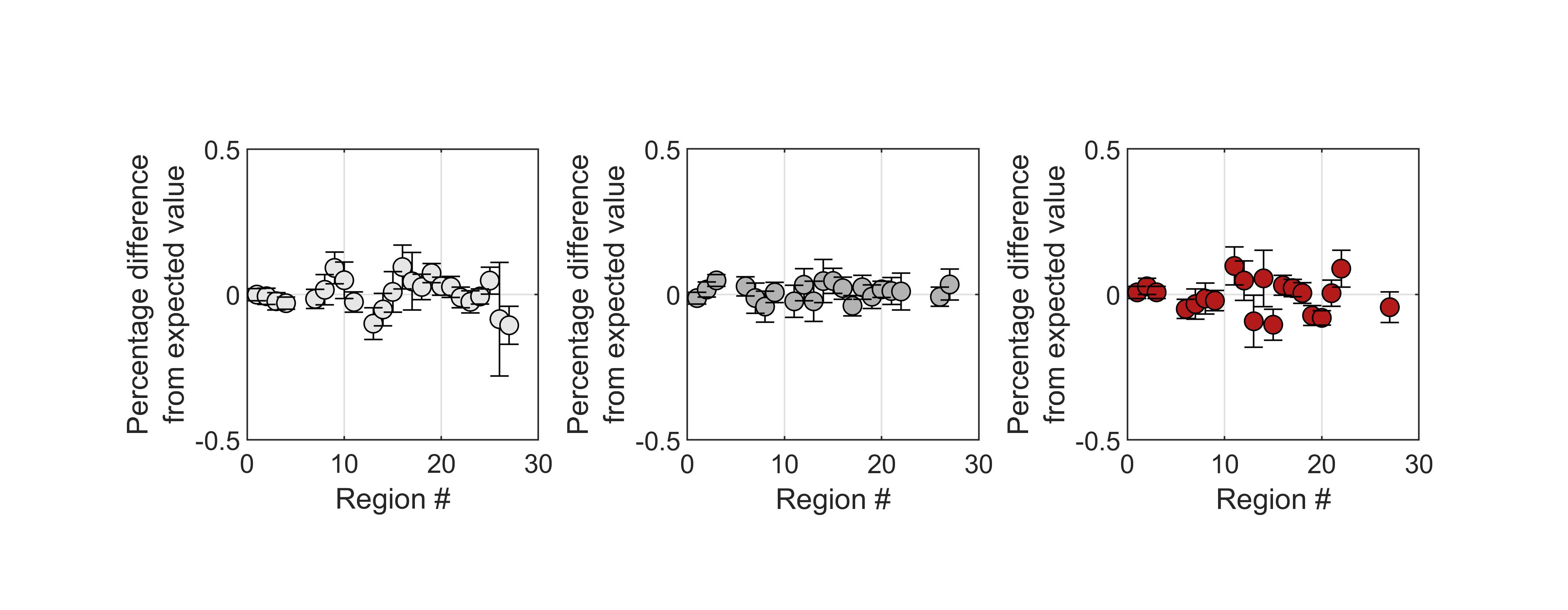

Supplement: Supplementary file 1 [file MP-47-3165-s001.jpg]
